# Effects of exercise interventions on breast cancer-related endogenous hormones in premenopausal and postmenopausal women: a systematic review and meta-analysis

**DOI:** 10.3389/fphys.2025.1579649

**Published:** 2025-09-26

**Authors:** Bo He, Xiaomei Yu

**Affiliations:** ^1^ School of Physical Education Chongqing, University of Posts and Telecommunications, Chongqing, China; ^2^ School of Physical Education and Health Management, Chongqing University of Education, Chongqing, China

**Keywords:** exercise intervention, breast cancer, sex hormones, postmenopausal, meta-analysis

## Abstract

**Background:**

Breast cancer is the most common malignant tumor among women, and its etiology and progression are closely associated with hormone levels. Hormone levels undergo significant changes in pre- and postmenopausal women. Exercise intervention, as a safe and effective lifestyle intervention, may modulate hormone levels and affect the incidence and prognosis of breast cancer.

**Methods:**

Three databases were searched to identify relevant literature for this study, which included 11 studies in the meta-analysis. The impact of an exercise intervention on breast cancer-related hormones was evaluated, including estrone, estradiol, free estradiol, testosterone, SHBG, 2-OH E1, 16a-OH E1, androstenedione, testosterone, and free testosterone, in both pre- and postmenopausal women.

**Results:**

The study findings suggest that the impact of exercise intervention on breast cancer-related hormones in pre- and postmenopausal women may not be significant. This lack of significance could be linked to differences in exercise intervention protocols, study quality, changes in body fat percentage post-exercise, and the specific characteristics of the populations (pre- and postmenopausal) analyzed in the studies. However, subgroup analyses suggested that exercise intervention might have a significant effect on certain estrogens in postmenopausal women and women who engaged in exercise for more than 6 months.

**Conclusion:**

The impact of exercise intervention on hormone levels may be influenced by body fat and menopausal status, as well as the duration of follow-up. Further high-quality and standardized studies are needed to confirm and enhance the findings of this research.

**Systematic Review Registration:**

https://www.crd.york.ac.uk/PROSPERO/view/CRD42024430643, Identifier CRD42024430643.

## 1 Introduction

Breast cancer is a significant and deadly form of malignancy that disproportionately affects women (Waks and Winer, 2019). The latest Cancer Statistics report from 2020 highlights that breast cancer accounts for 30% of all female cancer cases, with approximately 276,480 new diagnoses and over 42,000 fatalities in the same year (Siegel et al., 2020). Estrogen plays a critical role in the development and progression of breast cancer, serving as a key factor among various influencing elements (Burguin et al., 2021). Estrogen facilitates the initiation and progression of breast cancer by binding to estrogen receptors on breast cells, stimulating the proliferation and differentiation of breast cells, metabolizing estrogen into genotoxic compounds such as DNA adducts, and silencing tumor suppressor genes (TSGs) involved in the development of breast cancer by inducing hypermethylation of gene promoters (Coyle, 2008). Lowering estrogen levels is considered an effective strategy for preventing and treating breast cancer. Menopause marks a significant physiological transition for women, leading to a decrease in ovarian function and a substantial reduction in estrogen secretion by the ovaries. Despite this, other tissues such as adipose tissue and adrenal glands are still capable of synthesizing a certain amount of estrogen (Sipilä et al., 2013; Leite et al., 2010).

Multiple studies have shown that exercise is a beneficial strategy in the prevention and treatment of breast cancer. Exercise can impact estrogen levels in women through various mechanisms (Howden et al., 2019; Friedenreich et al., 2019; Oh et al., 2022), including reducing body weight and body fat percentage (Schwingshackl et al., 2013), enhancing insulin sensitivity (Roberts et al., 2013), modulating immune function (Neilson et al., 2009; Schmidt et al., 2017), and inhibiting aromatase activity (Paulo et al., 2019). Epidemiological studies have shown that regular physical activity reduces the risk of both premenopausal and postmenopausal breast cancer. Additionally, eliminating physical inactivity as a risk factor has the potential to prevent around 10% of breast cancer cases globally (Palesh et al., 2018). Clinical studies have shown that exercise interventions, when used as an adjuvant treatment, can improve fatigue, depression, and quality of life in breast cancer patients (Dieli-Conwri et al., 2018; Puklin et al., 2023; Soriano-Maldonado et al., 2019; Aydin et al., 2021). Additionally, these interventions can reduce the risk of recurrence and mortality, as well as improve survival (Cannioto et al., 2021). Mechanistic studies have indicated that exercise may inhibit breast cancer cell growth and tumor formation by elevating blood catecholamine levels and activating the Hippo signaling pathway (Dethlefsen et al., 2017). Systematic reviews have consistently shown that regardless of the specific exercise protocols utilized, the majority of studies have found a reduction in circulating levels of estrone and estradiol, as well as an increase in sex hormone-binding globulin (SHBG) following intentional exercise interventions (van Gemert et al., 2015; Brown et al., 2022). In the context of breast cancer research, hormones associated with breast cancer risk and development primarily include estrogens (estrone, estradiol, and free estradiol), sex hormone-binding globulin (SHBG), androgens (testosterone, androstenedione, and free testosterone), and estrogen metabolites (2-hydroxyestrone [2-OHE1] and 16α-hydroxyestrone [16α-OHE1]), which collectively influence the hormonal milieu that affects breast cancer pathogenesis. When comparing the effects of exercise on sex hormones with a non-exercising control group, inconsistent results were obtained (Gonzalo-Encabo et al., 2019). While the overall impact of physical activity on sex hormones linked to breast cancer in women has been validated, the risk of breast cancer in postmenopausal women is positively correlated with estrogen levels in the body, particularly in estrogen receptor-positive breast cancer patients. Several exercise intervention studies in premenopausal women have demonstrated that exercise can reduce circulating estrogen levels, lengthen menstrual cycles, and decrease the number of ovulations (Krishnan et al., 2014). The impact of exercise on breast cancer-related sex hormones in pre- and postmenopausal women remains uncertain, as indicated by inconclusive results (Robles Gil et al., 2012). The specific roles of weight loss and physical activity in regulating steroid hormones are not clearly understood, thus complicating the ability to provide definitive guidelines and recommendations for exercise interventions. To address this issue, the present study aims to use a systematic meta-analysis approach to standardize quality assessment, data extraction, and effect size calculation of existing randomized controlled studies on the effects of physical activity on breast cancer-related sex hormones in premenopausal and postmenopausal women. This will help in drawing more robust and valid conclusions.

## 2 Objects and methods

### 2.1 Literature search strategy

To systematically review the impact of exercise interventions on breast cancer-related sex hormones in postmenopausal and premenopausal women, we implemented the following literature search strategy. Initially, we delineated four key concepts derived from the research question: exercise, breast cancer, sex hormones, and menopause. Secondly, we conducted searches in the PubMed database using each of the four concepts as subject terms. We utilized the MeSH (Medical Subject Headings) database and reference lists from relevant literature to identify corresponding free terms for the subject terms. Thirdly, we employed the Boolean operator OR to merge the subject terms and free terms within each concept, creating four distinct search subsets. Finally, we utilized the Boolean operator AND to combine these four search subsets in order to acquire the ultimate search results. The search strategies employed for both the Web of Science and Embase databases were consistent. For detailed search strategies and results, please refer to the Appendix. PROSPERO registration number: 430,643.

### 2.2 Inclusion of eligibility criteria

This meta-analysis followed the PRISMA (Preferred Reporting Items for Systematic Reviews and Meta-Analyses) guidelines (Page et al., 2021). The inclusion criteria were established based on the PICOS (Population, Intervention, Comparison, Outcome, Study Design) model (Amir-Behghadami and Janati, 2020) (Table 1). The study population (P) included both postmenopausal and premenopausal women, as well as diseased versus non-diseased women to allow for comprehensive subgroup analyses. The intervention group (I) was defined as structured physical activity programs meeting the following minimum criteria: frequency (≥2 times per week), duration (≥30 min per session), intervention period (≥12 weeks), and intensity (moderate to vigorous physical activity). The control group (C) was restricted to participants who maintained their usual lifestyle without participating in any structured exercise programs during the study period. The outcome indicators (O) were categorized into primary indicators, including estradiol, estrone, Sex Hormone-Binding Globulin (SHBG), and free estradiol, and secondary indicators, such as 2-OH E1, 16a-OH E1, androstenedione, testosterone, and free testosterone. The study population was limited to adult women (≥18 years) participating in randomized controlled trials investigating exercise interventions and hormone-related outcomes. To ensure methodological rigor and data quality, the exclusion criteria for the studies included: 1) articles with incomplete data that hindered proper extraction, 2) non-human studies, 3) non-randomized controlled trials like case-control, single-group pre- and post-control, and cross-sectional studies, 4) studies lacking relevant outcome measures, and 5) non-original studies (e.g., letters, reviews, etc.).

**TABLE 1:** Basic characteristics of include studies.

Author + year	Region	Intervention	Pre/Post	Patients		Follow-up	Age		BMI		Body weight		Body fat	
				On	Off		On	Off	On	Off	On	Off	On	Off
Friedenreic et al. (2010)	Canada	Aerobic exercise	Post	152	153	12 months	61.2 (5.4)	60.6 (5.7)	29.1 (4.5)	29.2 (4.3)	−2.3 (3.7)	−0.5 (3.8)	—	—
Friedenreich et al. (2015)	Canada	Aerobic exercise	Post	198	189	12 months	59.4 (4.8)	59.5 (5.1)	29.1 (4.4)	29.4 (4.4)	−2.6 (4.9)	−1.9 (4.1)	−2.0 (3.5)	−1.1 (2.8)
Campbell et al. (2007)	Canada	Aerobic exercise	Pre	17	15	12 weeks	25.5 (4.5)	25.9 (5.0)	−0.2 (2.7)	0.2 (2.9)	−0.4 (7.4)	0.5 (10.2)	−0.8 (6.6)	0.8 (5.8)
Campbell et al. (2012)	Canada	Aerobic exercise	Post	117	87	12 months	58.1 (5.0)	57.4 (4.4)	30.7 (3.7)	30.7 (3.9)	−2.8 (12.3)	−0.5 (12.4)	−1.8 (4.6)	−0.1 (4.9)
Orsatti et al. (2008)	Brazil	Resistance training	Post	22	21	16 weeks	57.8 (8.0)	59.3 (6.2)	0.6 (0.9)	0.5 (0.99)	—	—	−0.7 (2.5)	−0.9 (1)
McTiernan et al. (2004)	United Kingdom	Aerobic exercise	Post	87	86	12 months	60.7 (6.7)	60.6 (6.8)	30.4 (4.1)	30.5 (3.7)	—	—	47.4 (4.8)	47.3 (4.6)
Gonzalo-Encabo et al. (2020)	Spain	Endurance training	Post	10	12	12 weeks	56.7 (3.01)	57.4 (5.03)	32.5 (5.1)	35.7 (5.9)	84.5 (15.7)	90.7 (9.6)	37.4 (12.6)	41.7 (9.6)
Krishnan et al. (2014)	United States	Aerobic exercise、Resistance training	Pre	18	10	6 months	—	—	—	—	—	—	—	—
Smith et al. (2011)	American	Aerobic exercise	Pre	166	153	16 weeks	25.4(3.4)	25.2(3.5)	−0.01 (0.77)	0.01 (0.74)	−0.03 (2.6)	0.03 (2.5)	−0.95 (2.6)	−0.09 (2.5)
Atkinson et al. (2004)	United States	Aerobic exercise	Post	87	86	12 months	60.7 (6.7)	60.6 (6.8)	30.4 (4.1)	30.5 (3.7)	81.4 (14.1)	81.7 (12.1)	47.5 (4.8)	47.4 (4.6)
Nuri et al., (2012)	Iran	Aerobic exercise、Resistance training	Post	15	14	15 weeks	—	—	0.56 (3.5)	−0.3 (4.7)	−0.99 (13.1)	1.43 (9.28)	—	—

Pre, premenopause; Post, postmenopause; on, Experimental group; off, control group.

BMI, Body Mass Index; USA, United States of America; UK, United Kingdom.

Data are presented as mean (standard deviation) where applicable.

“-” indicates data not reported or not applicable.

### 2.3 Study selection and data extraction

This meta-analysis utilized a meticulous framework for literature screening and data extraction to uphold research rigor and data reliability. During the initial screening phase, two independent reviewers (XM,Y and YY) assessed the eligible literature by examining the titles and abstracts against predefined inclusion criteria to eliminate literature that did not align with the study objective. Subsequently, the selected literature was reviewed in full text to evaluate compliance. Any discrepancies in the review were resolved with the assistance of a third expert, Cf,L to ensure objectivity and consistency in the review process. The data extraction phase included gathering basic literature information such as the first author, title, country, and year of publication, as well as baseline participant characteristics like age, menopausal status and duration, BMI, body weight, body fat percentage, and baseline physical activity level. It also involved collecting intervention details such as type, cycle, frequency, and duration, along with information on measurement tools and outcomes.

### 2.4 Assessment of bias

To ensure the reliability and validity of our findings, This study employed Review Manager 5.4.1 software for the independent evaluation of literature quality, which was carried out by two professional assessors. To minimize assessor bias, both reviewers were trained in the Cochrane risk of bias assessment tool prior to evaluation, and disagreements were resolved through discussion with a third expert. The evaluation criteria consisted of the generation of randomized sequences, allocation concealment, blinding of participants and researchers, blinding of outcome assessments, completeness of outcome data, selective reporting, and other potential biases. Each criterion was assessed as ‘low risk,’ ‘uncertain,’ or ‘high risk’ depending on the level of bias present. A third expert was consulted to resolve any disagreements in the evaluation. To quantify the study effect and synthesize the results, a meta-analysis was conducted using Review Manager 5.4.1 and Stata 15.1 software. The choice between fixed-effects and random-effects models was determined by both statistical heterogeneity (I^2^ statistic) and clinical heterogeneity assessment. A fixed-effects model was used when I^2^ was less than 50% and studies were clinically homogeneous, while a random-effects model was employed when I^2^ exceeded 50% or when significant clinical heterogeneity was present regardless of the I^2^ value. The standardized mean difference (SMD) was utilized as an indicator of the effect size for continuous outcome variables. Furthermore, funnel plots and Egger regression tests were employed to examine potential publication bias. When significant publication bias was detected, sensitivity analyses were conducted and the impact on result interpretation was explicitly discussed.

## 3 Results

### 3.1 Results of the literature search

**FIGURE 1:**
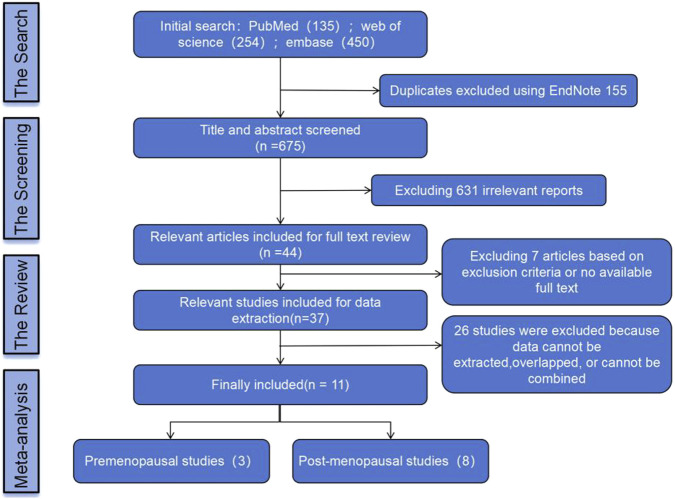
Literature screening process.

In this study, a total of 839 literature records were retrieved through database search and reference collection of relevant papers. After removing 155 duplicates, 675 records were screened. Non-RCT studies that were incomplete, duplicated, or irrelevant were excluded by reviewing the title, abstract, and full text. Ultimately, eleven eligible studies were included. The process of literature screening is outlined in Figure 1.

### 3.2 Study characterization and risk assessment

**FIGURE 2:**
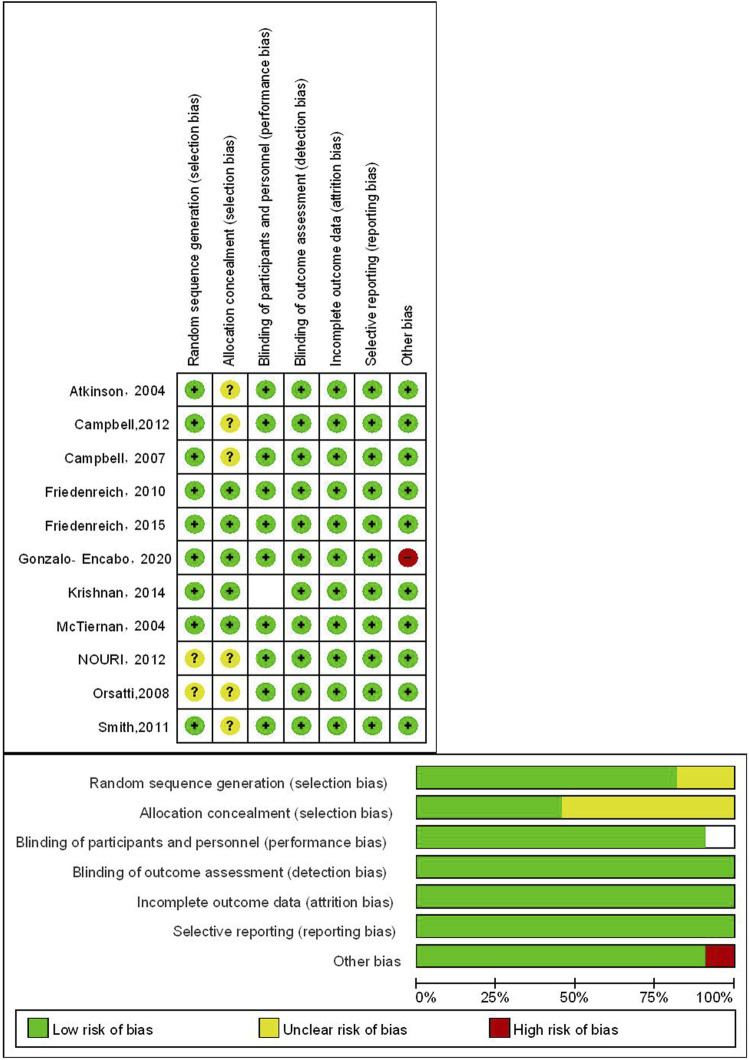
Risk of bias of the included studies.

Three studies included premenopausal women, while eight studies included postmenopausal women. The risk of bias assessment for the studies can be seen in Figure 2. All included studies demonstrated low to moderate risk of bias across key domains, with no studies classified as high risk in any critical bias category.

### 3.3 Meta-analysis results

#### 3.3.1 Main outcome indicators

**FIGURE 3:**
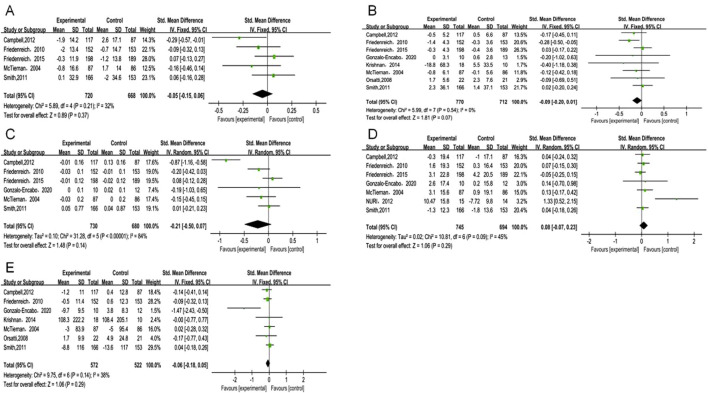
Forest plot of primary outcomes. **(A)** Estrone; **(B)** Estradiol; **(C)** Free estradiol; **(D)** SHBG; **(E)** Testosterone.

**FIGURE 4:**
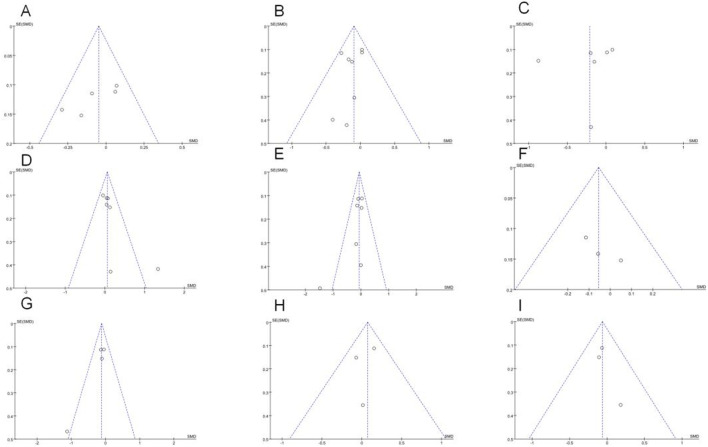
Funnel plots of **(A)** Estrone; **(B)** Estradiol; **(C)** Free estradiol; **(D)** SHBG; **(E)** Testosterone; **(F)** Androstenedione; **(G)** Free testosterone; **(H)** 2-OHE1;**(I)** 16α-OHE1.

Estrone analysis encompassed 5 studies with 1,388 participants (720 exercise, 668 control). Exercise intervention showed no significant effect (SMD = −0.05, 95% CI [−0.15, 0.06], p = 0.37) (Table 2), with low heterogeneity (I^2^ = 32%) (Figure 3A). Despite minor funnel plot asymmetry (Figure 4), statistical tests confirmed no publication bias (Egger’s p = 0.065, Begg’s p = 0.050).

**TABLE 2:** Subgroup analysis.

Subgroup	Estrone				Estradiol				SHBG				Testosterone			
	Study	SMD [95%CI]	*P*Value	*I* ^2^	Study	SMD [95%CI]	*P*Value	*I* ^2^	Study	SMD [95%CI]	*P*Value	*I* ^2^	Study	SMD [95%CI]	*P*Value	*I* ^2^
Total	5	−0.05 [−0.15, 0.06]	0.37	32%	8	−0.09 [−0.20, 0.01]	0.07	0%	7	0.08 [−0.07, 0.23]	0.29	45%	7	−0.06 [−0.18 ,0.05]	0.29	38%
Population																
premenopause	1	0.06 [−0.16, 0.28]	0.58	NA	2	−0.01 [−0.22, 0.2]	0.95	6%	1	0.04 [−0.18, 0.26]	0.78	NA	2	0.04 [−0.17, 0.25]	0.72	0%
post-menopause	4	−0.08 [−0.20, 0.04]	0.19	35%	6	−0.12 [−0.24, 0]	0.04	0%	6	0.11 [−0.09, 0.30]	0.27	54%	5	0.11 [−0.26, 0.03]	0.13	78%
Follow-up																
≥6 months	4	−0.08 [−0.20, 0.04]	0.19	35%	5	−0.13 [−0.25, −0.01]	0.04	11%	4	0.03 [−0.09, 0.15]	0.63	0%	4	−0.07 [−0.22, 0.07]	0.33	0%
<6 months	1	0.06 [−0.16, 0.28]	0.58	NA	3	0 [−0.2, 0.2]	0.99	0%	3	0.44 [−0.32, 1.21]	0.25	78%	3	−0.05 [−0.25, 0.15]	0.64	78%
Region																
Europe	1	−0.16 [−0.46, 0.14]	0.29	NA	2	−0.13 [−0.41, −0.15]	0.37	0%	2	0.13 [−0.15, 0.41]	0.38	0%	2	−0.11 [−0.39, 0.18]	0.46	88%
America	4	−0.03 [−0.14, 0.08]	0.58	43%	6	−0.09 [−0.20, −0.02]	0.11	15%	4	0.02 [−0.09, 0.13]	0.75	0%	5	−0.06 [−0.19, 0.08]	0.41	0%
Asia									1	1.36 [0.52, 2.15]	0.001	NA				

SMD, Standardized Mean Difference; CI, Confidence Interval; SHBG, Sex Hormone Binding Globulin.

I^2^, heterogeneity statistic; P-value, statistical significance level.

NA, Not applicable due to insufficient studies for analysis.

Estradiol meta-analysis included 8 studies (n = 1,482; 770 exercise, 712 control), revealing a borderline non-significant reduction (SMD = −0.09, 95% CI [−0.20, 0.01], p = 0.07) (Figure 3B). No heterogeneity was observed (I^2^ = 0%). Publication bias assessment showed no significant bias (Egger’s p = 0.065, Begg’s p = 0.050).

Free estradiol results from 6 studies (n = 1,410; 730 exercise, 680 control) demonstrated a larger magnitude of reduction that remained statistically non-significant (SMD = −0.21, 95% CI [−0.50, 0.07], p = 0.14) (Figure 3C). High heterogeneity (I^2^ = 84%) necessitated sensitivity analyses detailed below. Statistical evaluation revealed no publication bias (Egger’s p = 0.518, Begg’s p = 0.188) despite visual funnel plot asymmetry.

SHBG analysis comprised 6 studies (n = 1,266), while total testosterone encompassed 7 studies (n = 1,094; 572 exercise, 522 control). Exercise demonstrated non-significant trends toward increased SHBG (SMD = 0.08, 95% CI [−0.07, 0.23], p = 0.29) (Figure 3D) and decreased total testosterone (SMD = −0.06, 95% CI [−0.18, 0.05], p = 0.29) (Figure 3E). Moderate heterogeneity for SHBG (I^2^ = 45%) and low heterogeneity for total testosterone (I^2^ = 38%) supported result reliability. Publication bias tests showed no significant bias for either outcome (p ≥ 0.05).

#### 3.3.2 Secondary outcome indicators

**FIGURE 5:**
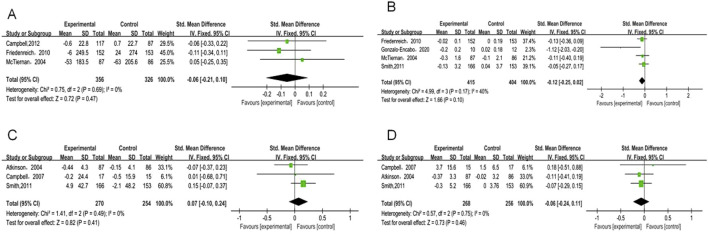
Forest plot of secondary outcomes **(A)** Androstenedione; **(B)** Free testosterone; **(C)** 2-OHE1; **(D)** 16α-OHE1.

Androstenedione analysis included 3 studies (n = 682; 356 exercise, 326 control), showing no significant intervention effect (SMD = −0.06, 95% CI [−0.21, 0.1], p = 0.47). No heterogeneity (I^2^ = 0%) or publication bias (p ≥ 0.05) was detected (Figure 5A).

Free testosterone analysis revealed no significant effect (SMD = −0.12, 95% CI [−0.25, 0.02], p = 0.1) (Figure 5B), while 2-OHE1 levels remained unchanged (SMD = 0.07, 95% CI [−0.1, 0.24], p = 0.41) (Figure 5C). Both outcomes showed complete homogeneity (I^2^ = 0%) and no publication bias (p ≥ 0.05). This lack of statistical significance may be due to These null findings may reflect insufficient intervention intensity or duration to influence these hormone metabolites associated with breast cancer risk.

16α-OHE1 data from 3 studies (n = 524; 268 exercise, 256 control) confirmed no significant intervention effects (SMD = −0.24, 95% CI [−0.24, 0.11], p = 0.46) with consistent results across studies (I^2^ = 0%) and no publication bias (p ≥ 0.05) (Figure 5D).

#### 3.3.3 Sensitivity analysis

**FIGURE 6:**
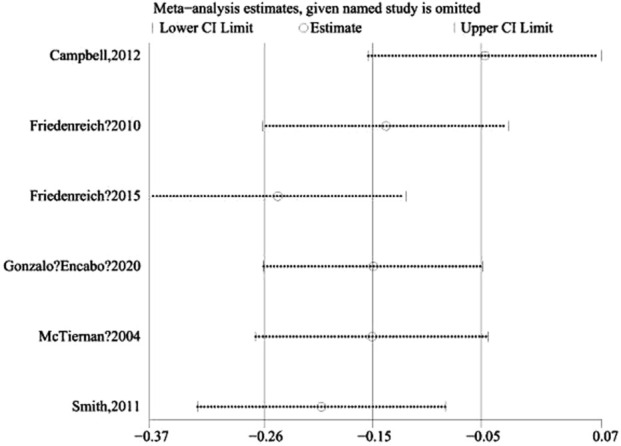
Heterogeneity test graph.

In this study, a sensitivity analysis was conducted on free estradiol using the one-by-one exclusion test. The results indicated a transition from non-significant to significant (SMD, 95% CI) for free estradiol upon exclusion of Campbell, 2012 (Figure 6), highlighting the high sensitivity of this outcome to that particular study.

#### 3.3.4 Subgroup analysis

The subgroup analysis results revealed that both the overall p-value of estrone and the p-values of each subgroup were greater than 0.05. Furthermore, the I^2^ values of each subgroup did not show a significant decrease compared to the overall I^2^ value. The studies included consistently showed that exercise intervention did not have a significant effect on estrone levels in women. However, for estradiol, the overall p-value was 0.07, with a p-value of 0.04 in the postmenopausal subgroup, indicating a potentially significant influence of exercise on estradiol levels in postmenopausal women, but not in premenopausal women. Given the imbalance in subgroup sizes and limited statistical power, these findings are hypothesis-generating rather than confirmatory. Furthermore, the subgroup with a follow-up duration of ≥6 months had a p-value of 0.04, suggesting that longer follow-up periods may lead to significant changes in estradiol levels. Formal tests for subgroup differences were not emphasized due to limited power and the imbalance between subgroups. Subgroup analysis of SHBG indicated a positive effect of exercise intervention on SHBG levels in a study conducted in Asia (p > 0.05), implying that exercise may not have a significant impact on testosterone levels. The observed variations in I^2^ values across subgroups suggest that heterogeneity may be influenced by factors such as geographic location, menopausal status, and follow-up duration. This emphasizes the significance of taking these variables into account when interpreting the effects of exercise intervention on endogenous hormones linked to breast cancer risk.

## 4 Discussion

Breast cancer, the most common cancer in women globally, is closely associated with hormone levels in the body. These hormone levels undergo significant changes as women transition from pre-to postmenopausal stages. Exercise interventions, considered a safe and effective lifestyle modification, have been hypothesized to potentially influence breast cancer risk through hormonal regulation, though the evidence remains inconclusive. This systematic meta-analysis, which included 11 randomized controlled trials, aimed to investigate the effects of exercise intervention on breast cancer-related hormones in both pre- and postmenopausal women. The main findings of this study are as follows:

The meta-analysis results suggest that exercise intervention may have a slight impact on reducing levels of estrone, estradiol, free estradiol, and testosterone, while increasing SHBG concentrations in pre- and postmenopausal women. Nevertheless, these effects were not statistically significant, indicating that the extent of these changes may be modest or vary significantly among individuals. Furthermore, the impact of exercise intervention on secondary outcome measures, such as 2-OH E1, 16a-OH E1, androstenedione, testosterone, and free testosterone, did not show significant changes. The results emphasize the intricate connection between exercise and hormonal regulation, emphasizing the necessity for additional research to clarify the potential mechanisms and pinpoint specific groups of women who could benefit the most from tailored exercise interventions.

The observed heterogeneity among the included studies may be attributed to several potential mechanisms. Firstly, the variability in intervention characteristics, such as exercise modality, intensity, frequency, and duration, could contribute to the inconsistent findings. The studies employed diverse exercise regimens, ranging from aerobic to resistance training, which may differentially influence hormonal responses. Moreover, participant adherence to the prescribed exercise protocols, even when similar in design, could introduce additional variability in the results. A systematic review has highlighted that the combination of endurance and resistance training may elicit a more pronounced reduction in estrogen expression compared to endurance training alone, underscoring the importance of exercise type in modulating hormonal profiles (Gonzalo-Encabo et al., 2019). This suggests that the type of exercise could play a crucial role in influencing estrogen expression. Furthermore, while the inclusion criteria of the studies were mostly similar, variations in baseline BMI and other characteristics among the study populations could potentially impact the levels of endogenous sex hormones. Thirdly, the studies varied in terms of the average weight loss observed in the intervention group. Studies have shown larger effects in those explicitly targeting weight loss (de Roon et al., 2018), with exercise-induced changes in estrogen levels in postmenopausal women often attributed to weight loss. Additionally (Friedenreich et al., 2011; Campbell et al., 2012; Stolzenberg-Solomon et al., 2012), BMI was found to be positively correlated with estrogen levels and negatively correlated with SHBG levels in women (Monninkhof et al., 2009). It was even noted that exercise interventions did not have a favorable effect on sex hormone levels in sedentary postmenopausal women. Women who lost more than 2% of their body fat experienced a decrease in the average levels of all estrogens and androgens. These studies indicate that exercise has a notable impact on the production of breast cancer-related sex hormones in both pre- and postmenopausal women, with body fat playing a key mediating role. Furthermore, a decrease in body fat percentage resulting from exercise can effectively suppress the production of breast cancer-related sex hormones. However, the study did not find a significant effect of exercise on breast cancer prevention when comparing the changes in hormones before and after the intervention between the exercise intervention group and the control group (Campbell et al., 2012; Monninkhof et al., 2009; Friedenreic et al., 2010).

The impact of exercise intervention on estrone and testosterone levels was consistent, with both the overall effect and subgroup analyses indicating that exercise intervention did not significantly affect the levels of estrone and testosterone. This finding aligns with previous studies (Krishnan et al., 2014; Monninkhof et al., 2009; Smith et al., 2011). McTiernan et al. (2006) (McTiernan et al., 2004) found that exercise intervention led to a notable reduction in serum estrone, estradiol, and free estradiol levels in both pre- and postmenopausal women. However, this effect was specifically observed in women with lower body fat. Accumulation of fat may contribute to insulin resistance, inflammation, and an imbalance in sex hormones, thereby elevating the risk of breast cancer diagnosis in postmenopausal women (Brown and Hankinson, 2015; Pacholczak et al., 2016). The impact of exercise intervention on estradiol levels may differ among subgroups, with postmenopausal women and those with a follow-up duration of 6 months or more potentially experiencing greater benefits. For example, exercise intervention has been shown to significantly reduce estradiol levels in postmenopausal women (Campbell et al., 2012; Friedenreic et al., 2010; McTiernan et al., 2004; Friedenreich et al., 2015; Orsatti et al., 2008). For premenopausal women, studies on the effects of exercise intervention found no significant differences in endogenous steroid hormone concentrations (estradiol, testosterone) within or between groups. There were also no significant interactions observed within or between groups (Krishnan et al., 2014). Moderate-intensity aerobic exercise without concurrent weight changes may not have a significant impact on reducing the risk of breast cancer (Smith et al., 2011). The impact of exercise on estradiol levels may be influenced by menopausal status and the duration of follow-up. Postmenopausal women primarily derive estrogen from adipose tissue due to decreased ovarian function, and exercise can lower estrogen synthesis by reducing adiposity. This, in turn, may help decrease the risk of breast cancer in postmenopausal women (Lizcano and Guzmán, 2014). Premenopausal women have normal ovarian function and primarily produce estrogen from the ovaries, so the impact of exercise on estrogen levels may be minimal or inconsistent. Furthermore, the duration of the follow-up period could also affect the relationship between exercise and estrogen; a longer follow-up period may reveal a more pronounced cumulative effect of exercise, potentially leading to a greater reduction in estrogen levels.

Third, the impact of exercise intervention on SHBG levels may vary by region. A study conducted in Asia found a significant increase in SHBG levels following exercise interventions (Nuri et al., 2012). Conversely, studies in other regions suggested that exercise interventions could potentially raise SHBG levels, but the overall effect was not statistically significant. This discrepancy in findings could be attributed to the lower baseline SHBG levels in Asian women and their dietary patterns. SHBG, a protein that binds estrogens and androgens, decreases free hormone levels, thus potentially reducing the risk of breast cancer (Dimou et al., 2019). Therefore, exercise may contribute to breast cancer prevention by elevating SHBG levels. However, SHBG levels are also influenced by other factors such as genetics, age, liver function, insulin, and thyroid hormones (Tymchuk et al., 2000). There may be variations in the impact of exercise on SHBG levels based on individual and geographical differences. It is possible that Asian women could exhibit a greater response to exercise compared to women in other regions, potentially due to genetic, dietary, and lifestyle factors, leading to notably higher levels of SHBG.

The limitations of this study include the small number of included studies and their low quality, which may introduce some risk of bias. Additionally, our search was restricted to English-language publications, which may have resulted in language bias and the exclusion of relevant studies published in other languages. The limited number of databases searched may have also contributed to potential selection bias. Variability in factors such as the type, intensity, frequency, and duration of exercise interventions across studies could impact the effectiveness of these interventions (Rinaldi et al., 2014). Hormone levels in pre- and postmenopausal women can be affected by various factors, including age, weight, diet, genetics, medications, etc. These factors were not adjusted or controlled for in the study, potentially leading to confounding or biased results. Furthermore, while statistical tests (Egger’s and Begg’s tests) indicated no significant publication bias for most outcomes, the visual asymmetry observed in some funnel plots suggests potential small-study effects that warrant consideration. Importantly, unequal subgroup sizes (fewer premenopausal than postmenopausal trials) and potential clinical heterogeneity restrict the interpretability of cross-group comparisons; accordingly, subgroup analyses in this review were descriptive rather than confirmatory, and any cross-group contrasts should be interpreted with caution. Hence, the findings of this research necessitate validation and refinement through additional, high-quality, standardized, and detailed studies. Future research should further explore whether exercise interventions can directly influence breast cancer occurrence and outcomes through hormonal pathways in both pre- and postmenopausal women. Additionally, it is essential to investigate the effects of exercise interventions on various biomarkers such as inflammatory factors, oxidative stress, and immune function that are associated with breast cancer. These studies aim to uncover the mechanisms underlying the effectiveness of exercise interventions and determine the most effective protocols for their application in the prevention and management of breast cancer.

## 5 Conclusion

This meta-analysis examined the impact of exercise intervention on breast cancer-related hormones in pre- and postmenopausal women. While no significant direct effect on individual hormones was identified, the majority of studies indicated an indirect influence through the reduction of body fat percentage, highlighting its role as a mediator in the relationship between exercise and hormonal changes. Subgroup analyses revealed more significant effects in postmenopausal women and participants with prolonged intervention adherence, emphasizing the crucial role of menopausal status and compliance in evaluating hormonal responses to exercise. However, no significant effect was observed on premenopausal women or those who participated in the intervention for less than 6 months. The results suggest that the effects of exercise interventions on sex hormones related to breast cancer in women may vary depending on factors such as menopausal status, body fat percentage, and duration of the intervention. Therefore, individualized, dynamic adaptation, and optimization of these interventions are necessary for different individuals and contexts.

## Data Availability

The original contributions presented in the study are included in the article/Supplementary Material, further inquiries can be directed to the corresponding author.
